# Hiding negative trials by pooling them: a secondary analysis of pooled-trials publication bias in FDA-registered antidepressant trials

**DOI:** 10.1017/S0033291718002805

**Published:** 2018-09-28

**Authors:** Ymkje Anna de Vries, Annelieke M. Roest, Erick H. Turner, Peter de Jonge

**Affiliations:** 1 Department of Psychiatry, Interdisciplinary Center Psychopathology and Emotion Regulation, University Medical Center Groningen, University of Groningen, Groningen, The Netherlands; 2 Developmental Psychology, Department of Psychology, University of Groningen, Groningen, The Netherlands; 3 Behavioral Health and Neurosciences Division, Portland Veterans Affairs Medical Center, Portland, OR, USA; 4 Department of Psychiatry, Oregon Health and Science University, Portland, OR, USA

**Keywords:** Antidepressants, bias, depression, pooled-trials publication bias

## Abstract

**Background:**

Previous studies on reporting bias generally examined whether trials were published in stand-alone publications. In this study, we investigated whether pooled-trials publications constitute a specific form of reporting bias. We assessed whether negative trials were more likely to be exclusively published in pooled-trials publications than positive trials and examined the research questions, individual trial results, and conclusions presented in these articles.

**Methods:**

Data from a cohort of 105 randomized controlled trials of 16 antidepressants were extracted from earlier publications and the corresponding Food and Drug Administration (FDA) reviews. A systematic literature search was conducted to identify pooled-trials publications.

**Results:**

We found 107 pooled-trials publications that reported results of 23 (72%) of 32 trials not published in stand-alone publications. Only two (3.8%) of 54 positive trials were published exclusively in pooled-trials publications, compared with 21 (41.1%) of 51 negative trials (*p* < 0.001). Thirteen (12%) of 107 publications had as primary aim to present data on the trial's primary research question (drug efficacy compared with placebo). Only four of these publications, reporting on five (22%) trials, presented individual efficacy data for the primary research question. Additionally, only five (5%) of 107 pooled-trials publications had a negative conclusion.

**Conclusions:**

Compared with positive trials, negative trials of antidepressants for depression were much more likely to be reported exclusively in pooled-trials publications. Pooled-trials publications flood the evidence base with often-redundant articles that, instead of addressing the original primary research question, present (positive) results on secondary questions. Therefore, pooled-trials publications distort the apparent risk–benefit profile of antidepressants.

## Introduction

Reporting bias has been demonstrated in many medical fields (McGauran *et al*., 2010; Hart *et al*., 2012; Dwan *et al*., 2013). An important form of reporting bias is study publication bias, which occurs when trials with positive results are more likely to be published than those with negative results (Higgins and Green, 2011). In studies on reporting bias, trials that are published exclusively in pooled-trials publications, which pool data from multiple trials, are usually regarded as unpublished (Turner *et al*., 2008, 2012) or incompletely published (Rising *et al*., 2008). However, some pharmaceutical companies have argued that these trials have actually been published (*Lilly press release* 2008).

Although pooled-trials publications can provide new information, they may be particularly susceptible to bias, for example, because it is often unclear how trials were selected for inclusion (Thaler *et al*., 2013). The research question of pooled-trials publications also often differs from the original research question. For example, they may focus on differential efficacy in patient subgroups, leading to substantial redundancy and the suggestion that many of these articles represent ‘salami publications’ (Spielmans *et al*., 2010, 2017). Additionally, negative trials, in contrast to positive trials, may be published exclusively in pooled-trials publications. A study examining trials for five antidepressants approved between 1989 and 1994 found that positive trials were usually reported in stand-alone publications, while negative trials were frequently ‘bundled’ into pooled-trials publications (Melander *et al*., 2003). Consequently, pooled-trials publications may actually further bias the published literature and the apparent risk–benefit profile of treatments, rather than helping to provide transparent access to trial results. However, that study (Melander *et al*., 2003) did not assess the research questions of pooled-trials publications, whether they were likely to reach positive conclusions, and whether individual trial results were also presented.

A previous meta-analysis found that 31% of antidepressant trials for major depressive disorder remained unpublished (Turner *et al*., 2008). However, pooled-trials publications were excluded from that study. In the present study, we use that meta-analysis and data from four novel antidepressants approved subsequently to investigate whether the practice of pooling trials constitutes a specific form of reporting bias. We assessed whether unpublished trials were actually published in pooled-trials publications and determined how frequently negative trials were published exclusively in pooled-trials publications compared with positive trials. Secondly, we evaluated whether the research question of pooled-trials publications corresponded to the original primary research question of the included trials and whether these publications reported individual trial results for this primary outcome. Finally, we assessed how often pooled-trials publications reached positive conclusions.

## Methods

### FDA-registered trials

Information on phase 2/3 clinical trial programs for 16 second-generation antidepressants (bupropion sustained release, citalopram, desvenlafaxine, duloxetine, escitalopram, fluoxetine, levomilnacipran, mirtazapine, nefazodone, paroxetine immediate release, paroxetine controlled release, sertraline, venlafaxine immediate release, venlafaxine extended release, vilazodone, and vortioxetine) was extracted from an earlier publication (Turner *et al*., 2008) or from Food and Drug Administration (FDA) reviews using the same methodology. These reviews can also be accessed online (Turner, 2013*a*; OHSU Digital Commons, n.d.). Because pharmaceutical companies must preregister trials they intend to conduct in support of US marketing approval, FDA reviews can be used as a registry and results database (Turner, 2004).

These programs included 105 trials investigating the short-term treatment of depression. Consistent with Turner *et al*. (2008), we extracted the FDA's regulatory decision [i.e. whether the primary endpoint(s) were judged to be positive]; 54 trials were considered positive and 51 trials not-positive in the current study. Detailed information on these trials is provided by Turner *et al*. (2008). We included only FDA-registered trials because this enabled us to assemble a complete cohort of premarketing trials; although some pharmaceutical companies have detailed trial registries (e.g. GlaxoSmithKline), it is generally difficult or impossible to obtain information on older, unpublished trials that are not FDA-registered.

### Trials published as stand-alone publications

We retrieved the references of 50 journal articles reporting the results of FDA-registered antidepressant trials from Turner *et al*. (2008). One article presented the pooled results of two identically designed trials of paroxetine controlled release (Golden *et al*., 2002); we regarded this article as a pooled-trials publication and the included trials were considered unpublished in stand-alone form. Additionally, we found a matching stand-alone publication (Miller *et al*., 1989) for one trial of paroxetine (UK-06) considered unpublished by Turner *et al.*, and we found 23 stand-alone articles reporting the results of novel antidepressant trials.

### Trials published in pooled-trials publications only

We assessed whether trials not published in stand-alone form were published in pooled-trials publications. Pooled-trials publications were defined as publications in which the individual patient data of two or more trials were analyzed. This included publications described as individual patient data meta-analyses, but it did not include meta-analyses based on aggregate data. A systematic literature search was conducted in PubMed, EMBASE, and the Cochrane Central Register of Controlled Trials, restricted to articles in English, until 15 August 2017. The search strategy included the drug, depression-related terms, and ‘placebo’. Terms were customized to each database; for example, the search string for searching PubMed for citalopram publications was: citalopram [Title] AND depress* [Title/abstract] AND placebo. After identifying pooled-trials publications, trial matches were identified using the drug name, active comparator (when applicable), dosage groups, sample sizes, trial duration, and names of investigators. We only included pooled-trials publications for which included trials could be matched to FDA-registered trials. From each publication, we extracted the primary endpoint, which was categorized as ‘primary efficacy’, i.e. the pooled-trials publication endpoint was the same as the original trial's primary endpoint (efficacy of the drug compared with placebo), or ‘not primary efficacy’. The second category consisted of secondary efficacy outcomes (e.g. anxiety, efficacy compared with an active comparator), predictors of efficacy (e.g. efficacy in subgroups, baseline severity), and other efficacy or safety outcomes. We also extracted whether publications reported individual trial results for the original primary outcome of the included trials. Additionally, AR and YV classified each pooled-trials publication as positive, neutral, or negative, based on the abstract (96% inter-rater agreement). Differences were resolved by consensus. Publications were considered positive when the abstract claimed that the antidepressant was more effective than placebo or an active comparator; equally effective as an active comparator; safer, better tolerated, or equal in safety/tolerability to placebo or an active comparator; or simply ‘safe’ or ‘well-tolerated’. Publications were considered neutral when the publication was primarily methodological in orientation or otherwise did not address the antidepressant in question.

### Statistical analysis

We examined whether not-positive trials were more likely to be published exclusively in pooled-trials publications than positive trials. Because of small cell sizes, *p* values were obtained with Fisher's exact test, using Stata (version 13.1).

## Results

### Pooled-trials publications

**Fig. 1.**
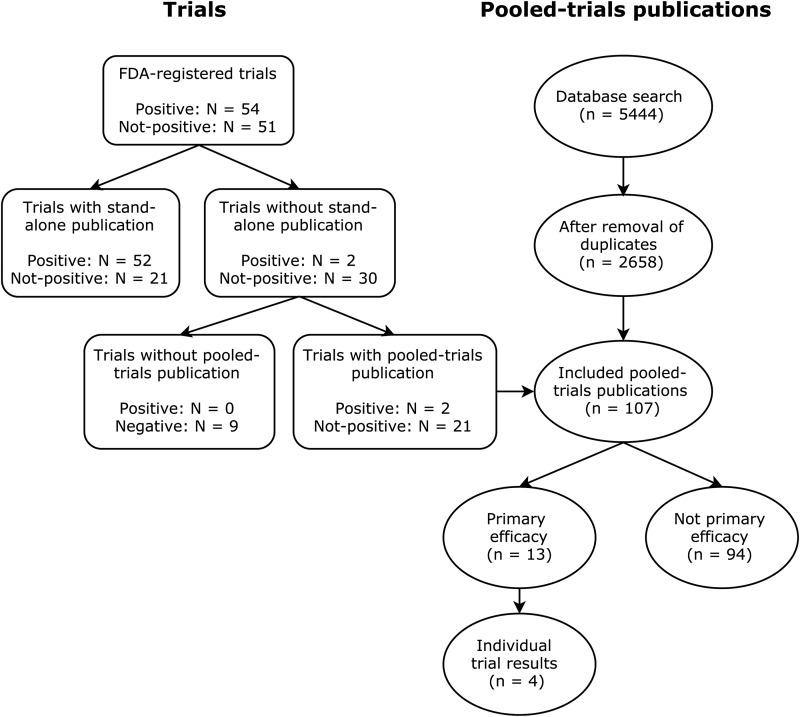
Flowchart of trial inclusion and pooled-trials publication inclusion.

As shown in the flow diagram (Fig. 1), of 105 FDA-registered antidepressant trials, 32 were not published in stand-alone publications. Of these, 23 (71.9%) were included in 107 pooled-trials publications (Table 1).

**Table 1.** Number of pooled-trials publications and their research questions for all trials not published in stand-alone form

Drug	Trial name	FDA decision	Number of pooled trials-publications	Primary efficacy as research question		Research question if not primary efficacy outcome			
				Total number	Individual trial results reported	Secondary efficacy outcomes	Predictors of efficacy	Other efficacy	Safety
Bupropion	205	Not positive	1	0	0	0	0	0	1
	212	Not positive	1	0	0	0	0	0	1
	Total		1	0	0	0	0	0	1
Citalopram	89306	Not positive	1	0	0	0	0	0	1
	Total		1	0	0	0	0	0	1
Desvenlafaxine	223	Not positive	11	1	0	3	1	1	5
	309	Not positive	10	2	1	3	1	0	4
	317	Not positive	10	2	1	3	1	0	4
	Total		12	2	1	3	1	1	5
Duloxetine	HMAT-A	Not positive	45	4	2	8	14	5	14
	HMAQ-B	Not positive	36	2	1	7	13	2	12
	Total		45	4	2	8	14	5	14
Escitalopram	SCT-MD-02	Not positive	16	0	0	6	4	4	2
	Total		16	0	0	6	4	4	2
Mirtazapine	003–020	Positive	4	0	0	3	0	1	0
	003–021	Not positive	4	0	0	3	0	1	0
	003–003	Not positive	2	0	0	1	0	1	0
	003–008	Not positive	2	0	0	1	0	1	0
	Total		4	0	0	3	0	1	0
Nefazodone	007	Not positive	0	0	0	0	0	0	0
	004A	Not positive	0	0	0	0	0	0	0
	Total		0	0	0	0	0	0	0
Paroxetine	01–001 (IR)	Not positive	2	0	0	0	0	0	2
	03–003 (IR)	Not positive	4	3	0	0	0	1	0
	07 (IR)	Not positive	0	0	0	0	0	0	0
	09 (IR)	Not positive	2	0	0	0	0	0	2
	UK-12 (IR)	Not positive	2	0	0	0	0	0	2
	448 (CR)	Not positive	2	1	0	0	1	0	0
	449 (CR)	Positive	2	1	0	0	1	0	0
	Total		8	4	0	0	1	1	2
Sertraline	315	Not positive	1	0	0	0	1	0	0
	101	Not positive	1	0	0	0	0	0	1
	310	Not positive	0	0	0	0	0	0	0
	Total		2	0	0	0	1	0	1
Venlafaxine	303 (IR)	Not positive	7	2	1	1	2	0	2
	367 (XR)	Not positive	13	2	0	6	3	1	1
	Total		18	3	1	7	5	1	2
Vilazodone	244	Not positive	0	0	0	0	0	0	0
	245	Not positive	0	0	0	0	0	0	0
	246	Not positive	0	0	0	0	0	0	0
	247	Not positive	0	0	0	0	0	0	0
	248	Not positive	0	0	0	0	0	0	0
	Total		0	0	0	0	0	0	0
All drugs	Grand total		107	13	4	27	26	13	28

CR, controlled release; FDA, Food and Drug Administration; IR, immediate release; XR, extended release.

**Fig. 2.**
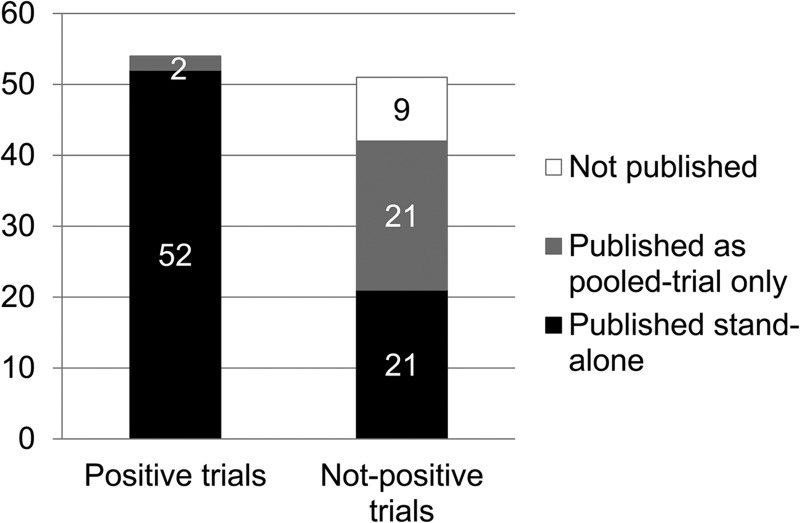
Publication status of positive and not-positive FDA trials.

Of the 51 not-positive trials, 21 (41.1%) were exclusively published in pooled-trials publications, compared with two (3.7%) positive trials (Fig. 2). Consequently, all positive trials were published in some form (either stand-alone or pooled), as were 82.4% of not-positive trials. Compared with positive trials, not-positive trials were more likely to be published exclusively in pooled-trials publications (Fisher's exact *p* < 0.001).

### Characteristics of pooled-trials publications

Thirteen of 107 (12.1%) pooled-trials publications, including 10 (43%) of 23 trials published exclusively in pooled-trials publications, had the same research question as the included trial's original primary research question (drug efficacy compared with placebo) (Table 1). Only four (3.7%) publications presented individual efficacy data for the primary research question, reporting efficacy results for five (21.7%) trials. Other publications reported on secondary efficacy outcomes (27 publications), predictors of efficacy (26 publications), other efficacy data (13 publications), or safety outcomes (28 publications) (Table 1).

Only five pooled-trials publications (4.7%) reported a negative conclusion (online Supplementary Table S1). One publication examined the general safety profile of duloxetine, two examined suicidality during paroxetine treatment, one examined discontinuation symptoms after stopping desvenlafaxine, and one examined the effect of desvenlafaxine on blood pressure. All five concluded that the drug was associated with more adverse events than placebo. Fourteen (13.1%) publications had neutral conclusions (predictors of efficacy = 5; other efficacy = 7; safety = 2) while the remaining 88 (82.2%) were positively framed.

## Discussion

### Main findings

To our knowledge, this study is the first to show that pooled-trials publication bias constitutes a specific form of reporting bias, which results in the publication of many positively framed articles supporting use of a drug, while the original, negative efficacy results of included trials remain obscured. Although 32 of 74 antidepressant trials were not published in stand-alone articles, only nine were completely unpublished, while the other 23 were included in pooled-trials publications. Trials lacking positive results were often exclusively published in pooled-trials publications (41.1%), whereas positive trials were usually published as stand-alone articles (96.3%). Importantly, only 12% of all pooled-trials publications [including 10 (43%) of 23 trials] examined the original primary research question (efficacy compared with placebo), and individual trial results for this original primary question were provided for only 22% of trials published exclusively in pooled-trials publications. Finally, only 5% of pooled-trials publications had a negative conclusion, all of which concerned safety. Therefore, although these trials have technically been published, the negative efficacy results are obscured, thus distorting the drugs’ apparent risk–benefit profile.

For some drugs, particularly duloxetine, the number of pooled-trials publications was very high. Trials HMAT-A and HMAQ-B were included in 44 and 36 publications, respectively. Our study thus replicates a prior report on the ‘salami slicing’ of duloxetine trials (Spielmans *et al*., 2010). We also found many pooled-trials publications for venlafaxine (18 publications for immediate- and extended-release combined) and escitalopram (16 publications). Several of these publications seemed redundant. For instance, three pooled-trials publications compared the efficacy of escitalopram to citalopram; three examined the effects of age and gender on the efficacy of venlafaxine; and eight compared the efficacy of venlafaxine to selective serotonin reuptake inhibitors.

It is noteworthy that duloxetine (approved in 2004) and escitalopram (approved in 2002) were the two newest antidepressants in Turner *et al.*’s (2008) meta-analysis, although venlafaxine ER was approved somewhat earlier in 1997. This suggests that the practice of pooling trials in many separate publications is relatively new, perhaps developing concurrently with physicians’ growing skepticism of advertising and sales representatives and greater trust in peer-reviewed publications (Spielmans and Parry, 2010). Consistent with this, a previous study examining antidepressants approved between 1989 and 1994 identified at most six pooled-trials publications for a single drug (Melander *et al*., 2003). Among novel antidepressants, we also found 12 pooled-trials publications for desvenlafaxine, but none for vilazodone. The latter may be because the unpublished vilazodone trials used a variety of dosages and sometimes used flexible dosages that included the approved dosage (20–40 mg), but also higher (e.g. 20–100 mg) or lower (e.g. 10–20 mg) doses.

Others have also noted that antidepressant meta-analyses (including pooled-trials publications) are massively produced, frequently have some kind of industry involvement, and rarely include negative statements (Ebrahim *et al*., 2015). In our study, 97 (91%) of 107 pooled-trials publications had at least one industry-employed author and, as noted, only five publications had negative conclusions. In all cases, this was because the antidepressant was associated with more adverse events than placebo, a finding that is not unexpected.

Although we cannot be certain of the intent behind these pooled-trials publications, the fact that they usually included industry employees as authors raises the question as to whether commercial interests may have played some role. In light of the growing concern that the medical literature may function as a marketing tool for pharmaceutical companies (Smith, 2005; Sismondo, 2009; The PLoS Medicine Editors, 2009; Spielmans and Parry, 2010; Vedula *et al*., 2012), pooled-trials publications may provide an easy and inexpensive way to keep a drug ‘in the spotlight’. Regardless of intent, the overall effect of these pooled-trials publications is to flood the evidence base with articles that encourage use of a drug, while discouraging results from the original trials remain invisible.

Many pooled-trials publications examined safety and tolerability. Because adverse events may occur infrequently, pooling trials can be necessary to achieve sufficient statistical power. The link between antidepressants and suicidality, for instance, was convincingly established only by pooling trials (Hammad *et al*., 2006; Stone *et al*., 2009). However, pooling can also mislead. For instance, bupropion SR was only approved at dosages of 300–400 mg/day, but a pooled-trials publication assessing its safety pooled all doses (50–400 mg/day) (Settle *et al*., 1999). Since adverse events are often dose-dependent, this is likely to paint an overly optimistic picture. Furthermore, there is ongoing concern that meta-analyses of harm outcomes may be particularly threatened by selective outcome reporting (Saini *et al*., 2014), a concern that is further increased by the possibility of selective trial inclusion in pooled-trials publications (Thaler *et al*., 2013).

### Limitations

Because some publications provided too little information for matching, we may have missed some relevant pooled-trials publications; however, individual trial results are never included in these publications. We specifically excluded 22 pooled-trials publications because it was unclear which trials were included. A second limitation is that we did not count pooled-trials publications that reported individual trial results for the original primary outcome but focused on a secondary research question, as it seemed unlikely that these results would be found by researchers or clinicians interested in the primary outcome. However, there were only five such publications, including three additional trials. Furthermore, most included trials were conducted prior to requirements for trial registration, and in the current study, more recent negative trials were more frequently published as stand-alone articles. However, older antidepressants are still in common use, so the results of these older trials continue to be relevant to clinicians. More subtle biases, such as spin or pooling trials, may also become more important as non-publication of a full trial becomes less common. We also did not conduct a meta-analysis, but focused on the *apparent* risk–benefit profile, following previous work in which the effect of reporting biases on apparent efficacy was larger than the effects on the meta-analytic effect size. For depression, for instance, the effect size of antidepressants was inflated from 0.31 to 0.41; while only 51% of trials were positive, 94% of published articles were positive (Turner *et al*., 2008). Finally, we assessed the presence of pooled-trials publication bias in a narrow field. However, such bias would be expected in other fields of medicine, because reporting bias has been found throughout psychiatry (Turner, 2013*b*; Le Noury *et al*., 2015; Roest *et al*., 2015), medicine (McGauran *et al*., 2010; Hart *et al*., 2012; Dwan *et al*., 2013), and science in general (Fanelli, 2012).

## Conclusions

Meta-analyses on reporting bias have been criticized by some for excluding pooled-trials publications, but our study shows that these publications are biased toward positive conclusions. As these publications rarely include individual trial results, they appear to serve primarily to heighten the (positive) visibility of a drug, rather than to transparently report negative results. Therefore, inclusion of pooled-trials publications in meta-analyses could lead to biased results, and caution is warranted. Pooled-trials publications may, however, also provide new information, for instance on secondary outcomes (Thaler *et al*., 2013). Ideally, individual patient data of all trials should be accessible so that biased reporting can no longer hamper the efforts of systematic reviewers. To mitigate the potential for bias, journal editors could also require pooled-trials publications to present individual trial results or reference stand-alone articles for all included trials. Additionally, editors, peer reviewers, and readers should be aware of the potential for bias and redundancy (Spielmans *et al*., 2010) and perhaps ask whether pooled-trials publications enhance or merely distort and bloat the evidence base.

In summary, the practice of pooling trials distorts the apparent risk–benefit profile of antidepressants by flooding the literature with publications that highlight positive results and obscure negative results. Together with study publication bias, selective outcome reporting, and spin, pooled-trials publication bias is a form of reporting bias that should be taken into account in future research.

## Supplementary material

For supplementary material accompanying this paper visit https://doi.org/10.1017/S0033291718002805. click here to view supplementary material
