# Supplementary material for: Hiding negative trials by pooling them: a secondary analysis of pooled-trials publication bias in FDA-registered antidepressant trials
Source: Psychol Med. 2018 Sep 28;49(12):2020–6. doi: 10.1017/S0033291718002805 (PMC6712952; doi:10.1017/S0033291718002805)
Supplement: Supplementary file 1 [file S0033291718002805sup001.docx]

**Supplemental table 1: Characteristics of included pooled-trials publications.**

| **Pooled–trials publication (author, year)** | **Unpublished trials included** | **Research question** | **Abstract judgement** |
| --- | --- | --- | --- |
|  | | | |
| **Bupropion** | | | |
| (1) Settle, 1999 | 205, 212 | Safety | Positive |
|  | | | |
| **Citalopram** | | | |
| (2) Pedersen, 2006 | 89306 | Safety | Positive |
|  | | | |
| **Desvenlafaxine** | | | |
| (3) Bech, 2010 | 223 | Other efficacy | Neutral |
| (4) Clayton, 2009 | 223, 309, 317 | Safety | Positive |
| (5) Kornstein, 2010 | 223, 309, 317 | Predictors of efficacy | Positive |
| (6) Kornstein, 2009 | 223, 309, 317 | Secondary efficacy | Positive |
| (7) ***Lieberman, 2008*** | ***309, 317*** | ***Primary efficacy*** | ***Positive*** |
| (8) Montgomery, 2009 | 223, 309, 317 | Safety | Negative |
| (9) Soares, 2009 | 223, 309, 317 | Secondary efficacy | Positive |
| (10) Thase, 2009 | 223, 309, 317 | Primary efficacy | Positive |
| (11) Thase, 2015 | 223 | Safety | Negative |
| (12) Tourian, 2010a | 223, 309, 317 | Secondary efficacy | Positive |
| (13) Tourian, 2010b | 223, 309, 317 | Safety | Positive |
| (14) Tourian, 2010c | 223, 309, 317 | Safety | Positive |
|  | | | |
| **Duloxetine** | | | |
| (15) Bailey, 2006 | HMAT-A, HMAQ-B | Predictors of efficacy | Positive |
| (16) Ball, 2011 | HMAT-A | Secondary efficacy | Positive |
| (17) Barros, 2014 | HMAT-A | Predictors of efficacy | Positive |
| (18) Bech, 2006 | HMAT-A | Other efficacy | Positive |
| (19) Brecht, 2008 | HMAT-A, HMAQ-B | Secondary efficacy | Positive |
| (20) Brunton, 2010 | HMAT-A, HMAQ-B | Safety | Negative |
| (21) Cookson, 2006 | HMAT-A, HMAQ-B | Secondary efficacy | Positive |
| (22) Delgado, 2005 | HMAT-A | Safety | Positive |
| (23) Dodd, 2014 | HMAT-A, HMAQ-B | Predictors of efficacy | Neutral |
| (24) Dodd, 2015 | HMAT-A, HMAQ-B | Safety | Neutral |
| (25) Dunner, 2003 | HMAT-A, HMAQ-B | Secondary efficacy | Positive |
| (26) Dunner, 2005 | HMAT-A, HMAQ-B | Safety | Positive |
| (27) Fishbain, 2008 | HMAT-A | Other efficacy | Positive |
| (28) Greist, 2004 | HMAT-A, HMAQ-B | Safety | Positive |
| (29) Gueorguieva, 2011 | HMAT-A, HMAQ-B | Primary efficacy | Positive |
| (30) Harada, 2015a | HMAT-A | Primary efficacy | Positive |
| (31) Harada, 2015b | HMAT-A, HMAQ-B | Secondary efficacy | Positive |
| (32) Harada, 2016 | HMAT-A, HMAQ-B | Safety | Neutral |
| (33) Hudson, 2005 | HMAT-A, HMAQ-B | Safety | Positive |
| (34) Kornstein, 2006 | HMAT-A, HMAQ-B | Predictors of efficacy | Positive |
| (35) Lewis-Fernandez, 2006 | HMAT-A, HMAQ-B | Predictors of efficacy | Positive |
| (36) Lobo, 2009 | HMAQ-B | Other efficacy | Neutral |
| (37) Mallinckrodt, 2003 | HMAT-A, HMAQ-B | Secondary efficacy | Positive |
| (38) Mallinckrodt, 2004 | HMAT-A, HMAQ-B | Other efficacy | Neutral |
| (39) Mallinckrodt, 2005 | HMAT-A, HMAQ-B | Predictors of efficacy | Positive |
| (40) ***Mallinckrodt, 2006*** | ***HMAT-A*** | ***Primary efficacy*** | ***Positive*** |
| (41) Mallinckrodt, 2008 | HMAT-A, HMAQ-B | Secondary efficacy | Positive |
| (42) Nelson, 2005 | HMAT-A, HMAQ-B | Predictors of efficacy | Positive |
| (43) Nelson, 2006 | HMAT-A | Safety | Positive |
| (44) Nelson, 2010 | HMAT-A, HMAQ-B | Predictors of efficacy | Positive |
| (45) Nelson, 2011 | HMAT-A, HMAQ-B | Predictors of efficacy | Neutral |
| (46) Nelson, 2013 | HMAT-A, HMAQ-B | Predictors of efficacy | Positive |
| (47) ***Nemeroff, 2002*** | ***HMAT-A, HMAQ-B*** | ***Primary efficacy*** | ***Positive*** |
| (48) Perahia, 2005 | HMAT-A, HMAQ-B | Safety | Positive |
| (49) Perahia, 2006 | HMAT-A, HMAQ-B | Predictors of efficacy | Positive |
| (50) Pritchett, 2007 | HMAT-A | Other efficacy | Positive |
| (51) Schacht, 2014 | HMAT-A, HMAQ-B | Predictors of efficacy | Neutral |
| (52) Shelton, 2007 | HMAT-A, HMAQ-B | Predictors of efficacy | Positive |
| (53) Stewart, 2006 | HMAT-A, HMAQ-B | Safety | Positive |
| (54) Thase, 2005 | HMAT-A, HMAQ-B | Safety | Positive |
| (55) Thase, 2007 | HMAT-A, HMAQ-B | Secondary efficacy | Positive |
| (56) Tokuoka, 2016 | HMAT-A, HMAQ-B | Predictors of efficacy | Neutral |
| (57) Viktrup, 2004 | HMAT-A, HMAQ-B | Safety | Positive |
| (58) Wernicke, 2007 | HMAT-A, HMAQ-B | Safety | Positive |
| (59) Wise, 2006 | HMAT-A, HMAQ-B | Safety | Positive |
|  | | | |
| **Escitalopram** | | | |
| (60) Baldwin, 2007 | SCT-MD-02 | Safety | Positive |
| (61) Baldwin, 2009 | SCT-MD-02 | Other efficacy | Neutral |
| (62) Bandelow, 2007 | SCT-MD-02 | Secondary efficacy | Positive |
| (63) Demyttenaere, 2008 | SCT-MD-02 | Secondary efficacy | Positive |
| (64) Demyttenaere, 2011 | SCT-MD-02 | Other efficacy | Neutral |
| (65) Gorman, 2002 | SCT-MD-02 | Secondary efficacy | Positive |
| (66) Kennedy, 2009 | SCT-MD-02 | Secondary efficacy | Positive |
| (67) Kilts, 2009 | SCT-MD-02 | Predictors of efficacy | Positive |
| (68) Lader, 2005 | SCT-MD-02 | Secondary efficacy | Positive |
| (69) Lam, 2006 | SCT-MD-02 | Predictors of efficacy | Positive |
| (70) Llorca, 2005 | SCT-MD-02 | Other efficacy | Positive |
| (71) Papakostas, 2011 | SCT-MD-02 | Predictors of efficacy | Positive |
| (72) Pedersen, 2005 | SCT-MD-02 | Safety | Positive |
| (73) Stein, 2006 | SCT-MD-02 | Other efficacy | Neutral |
| (74) Stein, 2011 | SCT-MD-02 | Secondary efficacy | Positive |
| (75) Wade, 2006 | SCT-MD-02 | Predictors of efficacy | Neutral |
|  | | | |
| **Mirtazapine** | | | |
| (76) Bech, 2001 | 003-020, 003-021 | Secondary efficacy | Positive |
| (77) Fawcett, 1998 | 003-003, 003-008, 003-020, 003-021 | Secondary efficacy | Positive |
| (78) Leucht, 2017 | 003-003, 003-008, 003-020, 003-021 | Other efficacy | Neutral |
| (79) Stahl, 1997 | 003-020, 003-021 | Secondary efficacy | Positive |
|  | | | |
| **Paroxetine IR and CR** | | | |
| (80) Carpenter, 2011 | 448, 449, UK-12, 01, 09 | Safety | Negative |
| (81) Dunbar, 1991 | 03-003 | Primary efficacy | Positive |
| (82) Feighner, 1992 | 03-003 | Primary efficacy | Positive |
| (83) Feighner, 1993 | 03-003 | Primary efficacy | Positive |
| (84) Kraus, 2010 | 448, 449, UK-12, 01, 09 | Safety | Negative |
| (85) Montgomery, 1992 | 03-003 | Other efficacy | Positive |
| (86) Dunner, 2005 | 448, 449 | Predictors of efficacy | Positive |
| (87) Golden, 2002 | 448, 449 | Primary efficacy | Positive |
|  | | | |
| **Sertraline** | | | |
| (88) Berti, 1995 | 315 | Predictors of efficacy | Positive |
| (89) Fisch, 1992 | 101 | Safety | Positive |
|  | | | |
| **Venlafaxine IR and XR** | | | |
| (90) Danjou, 1995 | 303 | Safety | Positive |
| (91) Entsuah, 1995a | 303 | Predictors of efficacy | Positive |
| (92) Entsuah, 1995b | 303 | Predictors of efficacy | Positive |
| (93) Entsuah, 2001 | 367 | Predictors of efficacy | Positive |
| (94) Entsuah, 2002 | 367 | Other efficacy | Positive |
| (95) Gibbons, 2012a | 303, 367 | Primary efficacy | Positive |
| (96) Gibbons, 2012b | 303, 367 | Safety | Positive |
| (97) Mallick, 2003 | 367 | Secondary efficacy | Positive |
| (98) ***Mendlewicz, 1995*** | ***303*** | ***Primary efficacy*** | ***Positive*** |
| (99) Nemeroff, 2008 | 367 | Secondary efficacy | Positive |
| (100) Rudolph, 1998 | 303 | Secondary efficacy | Positive |
| (101) Shelton, 2005 | 367 | Secondary efficacy | Positive |
| (102) Silverstone, 2002 | 367 | Predictors of efficacy | Positive |
| (103) Stahl, 2002 | 367 | Secondary efficacy | Positive |
| (104) Thase, 2001 | 367 | Secondary efficacy | Positive |
| (105) Thase, 2005 | 367 | Predictors of efficacy | Positive |
| (106) Thase, 2017 | 367 | Primary efficacy | Positive |
| (107) Trivedi, 2004 | 367 | Secondary efficacy | Positive |

Table notes: Studies that are in bold and italics include individual trial results.

**Full references**

1. Settle EC, Stahl SM, Batey SR, Johnston JA, Ascher JA. Safety profile of sustained-release bupropion in depression: Results of three clinical trials. Clin Ther. 1999 Mar;21(3):454–63.

2. Pedersen AG. Citalopram and suicidality in adult major depression and anxiety disorders. Nord J Psychiatry. 2006;60(5):392–9.

3. Bech P, Boyer P, Germain J-M, Padmanabhan K, Haudiquet V, Pitrosky B, et al. HAM-D17 and HAM-D6 sensitivity to change in relation to desvenlafaxine dose and baseline depression severity in major depressive disorder. Pharmacopsychiatry. 2010 Nov;43(7):271–6.

4. Clayton AH, Kornstein SG, Rosas G, Guico-Pabia C, Tourian KA. An integrated analysis of the safety and tolerability of desvenlafaxine compared with placebo in the treatment of major depressive disorder. CNS Spectr. 2009;14(4):183–95.

5. Kornstein SG, Clayton AH, Soares CN, Padmanabhan SK, Guico-Pabia CJ. Analysis by age and sex of efficacy data from placebo-controlled trials of desvenlafaxine in outpatients with major depressive disorder. J Clin Psychopharmacol. 2010 Jun;30(3):294–9.

6. Kornstein SG, Fava M, Jiang Q, Tourian KA. Analysis of depressive symptoms in patients with major depressive disorder treated with desvenlafaxine or placebo. Psychopharmacol Bull. 2009;42(3):21–35.

7. Lieberman DZ, Montgomery SA, Tourian KA, Brisard C, Rosas G, Padmanabhan K, et al. A pooled analysis of two placebo-controlled trials of desvenlafaxine in major depressive disorder. Int Clin Psychopharmacol. 2008;23(4):188–97.

8. Montgomery SA, Fava M, Padmanabhan SK, Guico-Pabia CJ, Tourian KA. Discontinuation symptoms and taper/poststudy-emergent adverse events with desvenlafaxine treatment for major depressive disorder. Int Clin Psychopharmacol. 2009 Nov;24(6):296–305.

9. Soares CN, Kornstein SG, Thase ME, Jiang Q, Guico-Pabia CJ. Assessing the efficacy of desvenlafaxine for improving functioning and well-being outcome measures in patients with major depressive disorder: A pooled analysis of 9 double-blind, placebo-controlled, 8-week clinical trials. J Clin Psychiatry. 2009 Oct;70(10):1365–71.

10. Thase ME, Kornstein SG, Germain J-M, Jiang Q, Guico-Pabia C, Ninan PT. An integrated analysis of the efficacy of desvenlafaxine compared with placebo in patients with major depressive disorder. CNS Spectr. 2009 Mar;14(3):144–54.

11. Thase ME, Fayyad R, Cheng R, Guico-Pabia CJ, Sporn J, Boucher M, et al. Effects of desvenlafaxine on blood pressure in patients treated for major depressive disorder: A pooled analysis. Curr Med Res Opin. 2015;31(4):809–20.

12. Tourian KA, Jiang Q, Ninan PT. Analysis of the effect of desvenlafaxine on anxiety symptoms associated with major depressive disorder: Pooled data from 9 short-term, double-blind, placebo-controlled trials. CNS Spectr. 2010 Mar;15(3):187–93.

13. Tourian KA, Leurent C, Graepel J, Ninan PT. Desvenlafaxine and weight change in major depressive disorder. Prim Care Companion J Clin Psychiatry. 2010;12(1):PCC.08m00746.

14. Tourian KA, Padmanabhan K, Groark J, Ninan PT. Retrospective analysis of suicidality in patients treated with the antidepressant desvenlafaxine. J Clin Psychopharmacol. 2010 Aug;30(4):411–6.

15. Bailey RK, Mallinckrodt CH, Wohlreich MM, Watkin JG, Plewes JM. Duloxetine in the treatment of major depressive disorder: comparisons of safety and efficacy. J Natl Med Assoc. 2006;98(3):437–47.

16. Ball SG, Desaiah D, Spann ME, Zhang Q, Russell JM, Robinson MJ, et al. Efficacy of duloxetine on painful physical symptoms in major depressive disorder for patients with clinically significant painful physical symptoms at baseline: A meta-analysis of 11 double-blind, placebo-controlled clinical trials. Prim Care Companion J Clin Psychiatry. 2011;13(6).

17. Barros BR, Schacht A, Happich M, Televantou F, Berggren L, Walker DJ, et al. Impact of pretreatment with antidepressants on the efficacy of duloxetine in terms of mood symptoms and functioning: An analysis of 15 pooled major depressive disorder studies. Prim Care Companion J Clin Psychiatry. 2014;16(5).

18. Bech P, Kajdasz DK, Porsdal V. Dose-response relationship of duloxetine in placebo-controlled clinical trials in patients with major depressive disorder. Psychopharmacology (Berl). 2006;188(3):273–80.

19. Brecht S, Kajdasz D, Ball S, Thase ME. Clinical impact of duloxetine treatment on sleep in patients with major depressive disorder. Int Clin Psychopharmacol. 2008;23(6):317–24.

20. Brunton S, Wang F, Edwards SB, Crucitti AS, Ossanna MJ, Walker DJ, et al. Profile of adverse events with duloxetine treatment: A pooled analysis of placebo-controlled studies. Drug Saf. 2010;33(5):393–407.

21. Cookson J, Gilaberte I, Desaiah D, Kajdasz DK. Treatment benefits of duloxetine in major depressive disorder as assessed by number needed to treat. Int Clin Psychopharmacol. 2006;21(5):267–73.

22. Delgado PL, Brannan SK, Mallinckrodt CH, Tran P V, McNamara RK, Wang F, et al. Sexual functioning assessed in 4 double-blind placebo- and paroxetine-controlled trials of duloxetine for major depressive disorder. J Clin Psychiatry. 2005 Jun;66(6):686–92.

23. Dodd S, Berk M, Kelin K, Zhang Q, Eriksson E, Deberdt W, et al. Application of the Gradient Boosted method in randomised clinical trials: Participant variables that contribute to depression treatment efficacy of duloxetine, SSRIs or placebo. J Affect Disord. 2014;168:284–93.

24. Dodd S, Schacht A, Kelin K, Duenas H, Reed VA, Williams LJ, et al. Nocebo effects in the treatment of major depression: Results from an individual study participant-level meta-analysis of the placebo arm of duloxetine clinical trials. J Clin Psychiatry. 2015 Jun;76(6):702–11.

25. Dunner DL, Goldstein DJ, Mallinckrodt C, Lu Y, Detke MJ. Duloxetine in treatment of anxiety symptoms associated with depression. Depress Anxiety. 2003;18(2):53–61.

26. Dunner DL, D’Souza DN, Kajdasz DK, Detke MJ, Russell JM. Is treatment-associated hypomania rare with duloxetine: Secondary analysis of controlled trials in non-bipolar depression. J Affect Disord. 2005;87(1):115–9.

27. Fishbain DA, Detke MJ, Wernicke J, Chappell AS, Kajdasz DK. The relationship between antidepressant and analgesic responses: findings from six placebo-controlled trials assessing the efficacy of duloxetine in patients with major depressive disorder. Curr Med Res Opin. 2008;24(11):3105–15.

28. Greist J, McNamara RK, Mallinckrodt CH, Rayamajhi JN, Raskin J. Incidence and duration of antidepressant-induced nausea: Duloxetine compared with paroxetine and fluoxetine. Clin Ther. 2004 Sep;26(9):1446–55.

29. Gueorguieva R, Mallinckrodt C, Krystal JH. Trajectories of depression severity in clinical trials of duloxetine: insights into antidepressant and placebo responses. Arch Gen Psychiatry. 2011 Dec 1;68(12):1227–37.

30. Harada E, Schacht A, Koyama T, Marangell LB, Tsuji T, Escobar R. Efficacy comparison of duloxetine and SSRIs at doses approved in Japan. Neuropsychiatr Dis Treat. 2015;11:115–23.

31. Harada E, Kato M, Fujikoshi S, Wohlreich MM, Berggren L, Tokuoka H. Changes in energy during treatment of depression: An analysis of duloxetine in double-blind placebo-controlled trials. Int J Clin Pract. 2015 Oct;69(10):1139–48.

32. Harada E, Shirakawa O, Satoi Y, Marangell LB, Escobar R. Treatment discontinuation and tolerability as a function of dose and titration of duloxetine in the treatment of major depressive disorder. Neuropsychiatr Dis Treat. 2016;12:89–97.

33. Hudson JI, Wohlreich MM, Kajdasz DK, Mallinckrodt CH, Watkin JG, Martynov O V. Safety and tolerability of duloxetine in the treatment of major depressive disorder: analysis of pooled data from eight placebo-controlled clinical trials. Hum Psychopharmacol Clin Exp. 2005;20(5):327–41.

34. Kornstein SG, Wohlreich MM, Mallinckrodt CH, Watkin JG, Stewart DE. Duloxetine efficacy for major depressive disorder in male vs. female patients: Data from 7 randomized, double-blind, placebo-controlled trials. J Clin Psychiatry. 2006 May;67(5):761–70.

35. Lewis-Fernandez R, Blanco C, Mallinckrodt CH, Wohlreich MM, Watkin JG, Plewes JM. Duloxetine in the treatment of major depressive disorder: comparisons of safety and efficacy in U.S. Hispanic and majority Caucasian patients. J Clin Psychiatry. 2006;67(0160–6689 (Print)):1379–90.

36. Lobo ED, Quinlan T, O’Brien L, Knadler MP, Heathman M, E.D. L, et al. Population pharmacokinetics of Orally administered duloxetine in patients: Implications for dosing recommendation. Clin Pharmacokinet. 2009;48(3):189–97.

37. Mallinckrodt CH, Goldstein DJ, Detke MJ, Lu Y, Watkin JG, Tran P V. Duloxetine: a new treatment for the emotional and physical symptoms of depression. Prim Care Companion J Clin Psychiatry. 2003 Feb;5(1):19–28.

38. Mallinckrodt CH, Raskin J, Wohlreich MM, Watkin JG, Detke MJ. The efficacy of duloxetine: a comprehensive summary of results from MMRM and LOCF_ANCOVA in eight clinical trials. BMC Psychiatry. 2004;4:26.

39. Mallinckrodt CH, Watkin JG, Liu C, Wohlreich MM, Raskin J. Duloxetine in the treatment of Major Depressive Disorder: a comparison of efficacy in patients with and without melancholic features. BMC Psychiatry. 2005;5:1.

40. Mallinckrodt CH, Prakash A, Andorn AC, Watkin JG, Wohlreich MM. Duloxetine for the treatment of major depressive disorder: A closer look at efficacy and safety data across the approved dose range. J Psychiatr Res. 2006 Jun;40(4):337–48.

41. Mallinckrodt CH, Prakash A, Houston JP, Swindle R, Detke MJ, Fava M. Differential antidepressant symptom efficacy: Placebo-controlled comparisons of duloxetine and SSRIs (fluoxetine, paroxetine, escitalopram). Neuropsychobiology. 2008;56:73–85.

42. Nelson JC, Wohlreich MM, Mallinckrodt CH, Detke MJ, Watkin JG, Kennedy JS. Duloxetine for the treatment of major depressive disorder in older patients. Am J Geriatr Psychiatry. 2005 Mar 1;13(3):227–35.

43. Nelson JC, Lu Pritchett Y, Martynov O, Yu JY, Mallinckrodt CH, Detke MJ. The safety and tolerability of duloxetine compared with paroxetine and placebo: a pooled analysis of 4 clinical trials. Prim Care Companion J Clin Psychiatry. 2006;8(4):212–9.

44. Nelson JC. Anxiety does not predict response to duloxetine in major depression: Results of a pooled analysis of individual patient data from 11 placebo-controlled trials. Depress Anxiety. 2010;27(1):12–8.

45. Nelson JC. Effects of baseline depression severity on remission rates with duloxetine and placebo in anxious and nonanxious patients with major depression. J Clin Psychopharmacol. 2011 Oct;31(5):682–4.

46. Nelson JC, Zhang Q, Kelin K, Eriksson E, Deberdt W, Berk M. Baseline patient characteristics associated with placebo remission and their impact on remission with duloxetine and selected SSRI antidepressants. Curr Med Res Opin. 2013 Jul;29(7):827–33.

47. Nemeroff CB, Schatzberg AF, Goldstein DJ, Detke MJ, Mallinckrodt C, Lu Y, et al. Duloxetine for the treatment of major depressive disorder. Psychopharmacol Bull. 2002;36(4):106–32.

48. Perahia DG, Kajdasz DK, Desaiah D, Haddad PM. Symptoms following abrupt discontinuation of duloxetine treatment in patients with major depressive disorder. J Affect Disord. 2005;89(1–3):207–12.

49. Perahia DGS, Kajdasz DK, Royer MG, Walker DJ, Raskin J. Duloxetine in the treatment of major depressive disorder: an assessment of the relationship between outcomes and episode characteristics. Int Clin Psychopharmacol. 2006 Sep;21(5):285–95.

50. Pritchett YL, Marciniak MD, Corey-Lisle PK, Berzon RA, Desaiah D, Detke MJ. Use of effect size to determine optimal dose of duloxetine in major depressive disorder. J Psychiatr Res. 2007;41:311–8.

51. Schacht A, Gorwood P, Boyce P, Schaffer A, Picard H. Depression symptom clusters and their predictive value for treatment outcomes: results from an individual patient data meta-analysis of duloxetine trials. J Psychiatr Res. 2014 Jun;53(1):54–61.

52. Shelton RC, Andorn AC, Mallinckrodt CH, Wohlreich MM, Raskin J, Watkin JG, et al. Evidence for the efficacy of duloxetine in treating mild, moderate, and severe depression. Int Clin Psychopharmacol. 2007;22:348–55.

53. Stewart DE, Wohlreich MM, Mallinckrodt CH, Watkin JG, Kornstein SG. Duloxetine in the treatment of major depressive disorder: Comparisons of safety and tolerability in male and female patients. J Affect Disord. 2006 Aug;94(1–3):183–9.

54. Thase ME, Tran P V, Wiltse C, Pangallo BA, Mallinckrodt C, Detke MJ. Cardiovascular profile of duloxetine, a dual reuptake inhibitor of serotonin and norepinephrine. J Clin Psychopharmacol. 2005;25(2):132–40.

55. Thase ME, Pritchett YL, Ossanna MJ, Swindle RW, Xu J, Detke MJ. Efficacy of duloxetine and selective serotonin reuptake inhibitors: comparisons as assessed by remission rates in patients with major depressive disorder. J Clin Psychopharmacol. 2007 Dec;27(6):672–6.

56. Tokuoka H, Takahashi H, Ozeki A, Kuga A, Yoshikawa A, Tsuji T, et al. Trajectories of depression symptom improvement and associated predictor analysis: An analysis of duloxetine in double-blind placebo-controlled trials. J Affect Disord. 2016 May;196:171–80.

57. Viktrup L, Pangallo BA, Detke MJ, Zinner NR. Urinary side effects of duloxetine in the treatment of depression and stress urinary incontinence. Prim Care Companion J Clin Psychiatry. 2004;6(2):65–73.

58. Wernicke J, Lledo A, Raskin J, Kajdasz DK, Wang F. An evaluation of the cardiovascular safety profile of duloxetine. Drug Saf. 2007;30(5):437–55.

59. Wise TN, Perahia DGS, Pangallo BA, Losin WG, Wiltse CG. Effects of the antidepressant duloxetine on body weight: analyses of 10 clinical studies. Prim Care Companion J Clin Psychiatry. 2006;8(5):269–78.

60. Baldwin DS, Reines EH, Guiton C, Weiller E. Escitalopram therapy for major depression and anxiety disorders. Ann Pharmacother. 2007;41(10):1583–92.

61. Baldwin DS, Stein DJ, Dolberg OT, Bandelow B. How long should a trial of escitalopram treatment be in patients with major depressive disorder, generalised anxiety disorder or social anxiety disorder? An exploration of the randomised controlled trial database. Hum Psychopharmacol Clin Exp. 2009 Jun;24(4):269–75.

62. Bandelow B, Andersen HF, Dolberg OT. Escitalopram in the treatment of anxiety symptoms associated with depression. Depress Anxiety. 2007;24:53–61.

63. Demyttenaere K, Andersen HF, Reines EH. Impact of escitalopram treatment on Quality of Life Enjoyment and Satisfaction Questionnaire scores in major depressive disorder and generalized anxiety disorder. Int Clin Psychopharmacol. 2008 Sep;23(5):276–86.

64. Demyttenaere K, Reines EH, Lönn SL, Lader M. Satisfaction with medication is correlated with outcome but not persistence in patients treated with placebo, escitalopram, or serotonin-norepinephrine reuptake inhibitors: a post hoc analysis. Prim care companion CNS Disord. 2011;13(4).

65. Gorman JM, Korotzer A, Su G. Efficacy comparison of escitalopram and citalopram in the treatment of major depressive disorder: pooled analysis of placebo-controlled trials. CNS Spectr. 2002 Apr;7(4 SUPPL. 1):40–4.

66. Kennedy SH, Andersen HF, Thase ME. Escitalopram in the treatment of major depressive disorder: A meta-analysis. Curr Med Res Opin. 2009 Jan 8;25(1):161–75.

67. Kilts CD, Wade AG, Andersen HF, Schlaepfer TE. Baseline severity of depression predicts antidepressant drug response relative to escitalopram. Expert Opin Pharmacother. 2009;10(6):927–36.

68. Lader M, Andersen HF, Bækdal T. The effect of escitalopram on sleep problems in depressed patients. Hum Psychopharmacol. 2005 Jul;20(5):349–54.

69. Lam RW, Andersen HF. The influence of baseline severity on efficacy of escitalopram and citalopram in the treatment of major depressive disorder: An extended analysis. Pharmacopsychiatry. 2006;39(5):180–4.

70. Llorca PM, Azorin JM, Despiegel N, Verpillat P. Efficacy of escitalopram in patients with severe depression: A pooled analysis. Int J Clin Pract. 2005;59(3):268–75.

71. Papakostas GI, Larsen K. Testing anxious depression as a predictor and moderator of symptom improvement in major depressive disorder during treatment with escitalopram. Eur Arch Psychiatry Clin Neurosci. 2011;261:147–56.

72. Pedersen AG. Escitalopram and suicidality in adult depression and anxiety. Int Clin Psychopharmacol. 2005;20:139–43.

73. Stein DJ, Baldwin DS, Dolberg OT, Despiegel N, Bandelow B. Which factors predict placebo response in anxiety disorders and major depression? An analysis of placebo-controlled studies of escitalopram. J Clin Psychiatry. 2006 Nov;67(11):1741–6.

74. Stein DJ, Lopez AG. Effects of escitalopram on sleep problems in patients with major depression or generalized anxiety disorder. Adv Ther. 2011;28(11):1021–37.

75. Wade A, Friis Andersen H. The onset of effect for escitalopram and its relevance for the clinical management of depression. Curr Med Res Opin. 2006;22(11):2101–10.

76. Bech P. Meta-analysis of placebo-controlled trials with mirtazapine using the core items of the Hamilton Depression Scale as evidence of a pure antidepressive effect in the short-term treatment of major depression. Int J Neuropsychopharmacol. 2001;4:337–45.

77. Fawcett J, Barbin RL. A meta-analysis of eight randomized, double-blind, controlled clinical trials of mirtazapine for the treatment of patients with major depression and symptoms of anxiety. J Clin Psychiatry. 1998;59(3):123–7.

78. Leucht S, Fennema H, Engel RR, Kaspers-Janssen M, Lepping P, Szegedi A. What does the MADRS mean? Equipercentile linking with the CGI using a company database of mirtazapine studies. J Affect Disord. 2017 Mar;210:287–93.

79. Stahl S, Zivkov M, Reimitz PE, Panagides J, Hoff W. Meta-analysis of randomized, double-blind, placebo-controlled, efficacy and safety studies of mirtazapine versus amitriptyline in major depression. Acta Psychiatr Scand. 1997;96(suppl 391):22–30.

80. Carpenter DJ, Fong R, Kraus JE, Davies JT, Moore C, Thase ME. Meta-analysis of efficacy and treatment-emergent suicidality in adults by psychiatric indication and age subgroup following initiation of paroxetine therapy: A complete set of randomized placebo-controlled trials. J Clin Psychiatry. 2011;72(11):1503–14.

81. Dunbar GC, Cohn JB, Fabre LF, Feighner JP, Fieve RR, Mendels J, et al. A comparison of paroxetine, imipramine and placebo in depressed out-patients. Br J Psychiatry. 1991 Sep;159:394–8.

82. Feighner JP. A double-blind comparison of paroxetine, imipramine and placebo in depressed outpatients. Int Clin Psychopharmacol. 1992 Jun;6 Suppl 4(suppl 4):31–5.

83. Feighner JP, Cohn JB, Fabre LF, Fieve RR, Mendels J, Shrivastava RK, et al. A study comparing paroxetine placebo and imipramine in depressed patients. J Affect Disord. 1993 Jun;28(2):71–9.

84. Kraus JE, Horrigan JP, Carpenter DJ, Fong R, Barrett PS, Davies JT. Clinical features of patients with treatment-emergent suicidal behavior following initiation of paroxetine therapy. J Affect Disord. 2010;120:40–7.

85. Montgomery SA. The advantages of paroxetine in different subgroups of depression. Int Clin Psychopharmacol. 1992 Jun;6 Suppl 4:91–100.

86. Dunner DL, Lipschitz A, Pitts CD, Davies JT. Efficacy and tolerability of controlled-release paroxetine in the treatment of severe depression: post hoc analysis of pooled data from a subset of subjects in four double-blind clinical trials. Clin Ther. 2005 Dec;27(12):1901–11.

87. Golden RN, Nemeroff CB, McSorley P, Pitts CD, Dubé EM. Efficacy and tolerability of controlled-release and immediate-release paroxetine in the treatment of depression. J Clin Psychiatry. 2002 Jul;63(7):577–84.

88. Berti C, Doogan DP, Scott NR, Dinan TG. Sertraline in the treatment of depressive disorders with associated anxiety. J Serotonin Res. 1995;3:151–70.

89. Fisch C, Knoebel SB. Electrocardiographic findings in sertraline depression trials. Drug Investig. 1992;4(4):305–12.

90. Danjou P, Hackett D. Safety and tolerance profile of venlafaxine. Int Clin Psychopharmacol. 1995 Mar;10 Suppl 2:15–20.

91. Entsuah R, Upton G V, Rudolph R. Efficacy of venlafaxine treatment in depressed patients with psychomotor retardation or agitation: a meta-analysis. Hum Psychopharmacol. 1995;10:195–200.

92. Entsuah AR, Rudolph RL, Chitra R. Effectiveness of venlafaxine treatment in a broad spectrum of depressed patients: a meta-analysis. Psychopharmacol Bull. 1995;31(4):759–66.

93. Entsuah AR, Huang H, Thase ME. Response and remission rates in different subpopulations with major depressive disorder administered venlafaxine, selective serotonin reuptake inhibitors, or placebo. J Clin Psychiatry. 2001;62(11):869–77.

94. Entsuah R, Gao B. Global benefit-risk evaluation of antidepressant action: comparison of pooled data for venlafaxine, SSRIs, and placebo. CNS Spectr. 2002 Dec;7(12):882–8.

95. Gibbons RD, Hur K, Brown CH, Davis JM, Mann JJ. Benefits from antidepressants: synthesis of 6-week patient-level outcomes from double-blind placebo-controlled randomized trials of fluoxetine and venlafaxine. Arch Gen Psychiatry. 2012 Jun;69(6):572–9.

96. Gibbons RD, Brown CH, Hur K, Davis J, Mann JJ. Suicidal thoughts and behavior with antidepressant treatment: reanalysis of the randomized placebo-controlled studies of fluoxetine and venlafaxine. Arch Gen Psychiatry. 2012 Jun;69(6):580–7.

97. Mallick R, Chen J, Entsuah AR, Schatzberg AF. Depression-free days as a summary measure of the temporal pattern of response and remission in the treatment of major depression: A comparison of venlafaxine, selective serotonin reuptake inhibitors, and placebo. J Clin Psychiatry. 2003;64(3):321–30.

98. Mendlewicz J. Pharmacologic profile and efficacy of venlafaxine. Int Clin Psychopharmacol. 1995 Mar;10 Suppl 2:5–13.

99. Nemeroff CB, Entsuah R, Benattia I, Demitrack M, Sloan DM, Thase ME. Comprehensive analysis of remission (COMPARE) with venlafaxine versus SSRIs. Biol Psychiatry. 2008;63(4):424–34.

100. Rudolph RL, Entsuah R, Chitra R. A meta-analysis of the effects of venlafaxine on anxiety associated with depression. J Clin Psychopharmacol. 1998 Apr;18(2):136–44.

101. Shelton C, Entsuah R, Padmanabhan SK, Vinall PE. Venlafaxine XR demonstrates higher rates of sustained remission compared to fluoxetine, paroxetine or placebo. Int Clin Psychopharmacol. 2005;20(4):233–8.

102. Silverstone PH, Entsuah R, Hackett D. Two items on the Hamilton Depression rating scale are effective predictors of remission: comparison of selective serotonin reuptake inhibitors with the combined serotonin/norepinephrine reuptake inhibitor, venlafaxine. Int Clin Psychopharmacol. 2002;17:273–80.

103. Stahl SM, Entsuah R, Rudolph RL. Comparative efficacy between venlafaxine and SSRIs: a pooled analysis of patients with depression. Biol Psychiatry. 2002;52(12):1166–74.

104. Thase ME, Entsuah AR, Rudolph RL. Remission rates during treatment with venlafaxine or selective seretonin reuptake inhibitors. Br J Psychiatry. 2001;178(MARCH.):234–41.

105. Thase ME, Entsuah R, Cantillon M, Kornstein SG. Relative antidepressant efficacy of venlafaxine and SSRIs: sex-age interactions. J women’s Heal. 2005 Sep;14(7):609–16.

106. Thase M, Asami Y, Wajsbrot D, Dorries K, Boucher M, Pappadopulos E. A meta-analysis of the efficacy of venlafaxine extended release 75–225 mg/day for the treatment of major depressive disorder. Curr Med Res Opin. 2017 Feb;33(2):317–26.

107. Trivedi MH, Wan GJ, Mallick R, Chen J, Casciano R, Geissler EC, et al. Cost and effectiveness of venlafaxine extended-release and selective serotonin reuptake inhibitors in the acute phase of outpatient treatment for major depressive disorder. J Clin Psychopharmacol. 2004;24(5):497–506.
